# X-ray Characterizations of Exfoliated MoS_2_ Produced by Microwave-Assisted Liquid-Phase Exfoliation

**DOI:** 10.3390/ma17163887

**Published:** 2024-08-06

**Authors:** Sebastiano Vasi, Salvatore Vincenzo Giofrè, Siglinda Perathoner, Domenico Mallamace, Salvatore Abate, Ulderico Wanderlingh

**Affiliations:** 1 Department of Mathematical and Computational Science, Physical Science and Earth Science, University of Messina, Viale F. Stagno D’Alcontres 31, I-98166 Messina, Italy; ulderico.wanderlingh@unime.it; 2 Department of Chemical, Biological, Pharmaceutical and Environmental Sciences, University of Messina, Viale Ferdinando Stagno D’Alcontres 31, I-98166 Messina, Italy; salvatorevincenzo.giofre@unime.it (S.V.G.); siglinda.perathon@unime.it (S.P.); d.mallamace@unime.it (D.M.); salvatore.abate@unime.it (S.A.)

**Keywords:** MoS2, molybdenum disulfide, liquid-phase exfoliation, microwave, XRD, XPS, 2D

## Abstract

An X-ray analysis of exfoliated MoS_2_, produced by means of microwave-assisted liquid-phase exfoliation (LPE) from bulk powder in 1-methyl-2-pyrrolidone (NMP) or acetonitrile (ACN) + 1-methyl-2-pyrrolidone (NMP) solvents, has revealed distinct structural differences between the bulk powder and the microwave-exfoliated samples. Specifically, we performed X-ray diffraction (XRD) and X-ray photoelectron spectroscopy (XPS) measurements to identify the elements of our exfoliated sample deposited on a Si substrate by drop-casting, as well as their chemical state and its structural crystalline phase. In the exfoliated sample, the peaks pattern only partially resemble the theoretical Miller indices for MoS_2_. In contrast, the bulk powder’s spectrum shows the characteristic peaks of the 2H polytype of MoS_2_, but with some broadening. Notable is the retention of partial crystallinity in the post-exfoliation phases, specifically in the normal-to-plane orientation, thus demonstrating the effectiveness of microwave-assisted techniques in producing 2D MoS_2_ and attaining desirable properties for the material. XPS measurements confirm the success of the exfoliation procedure and that the exfoliated sample retains its original structure. The exfoliation process has been optimized to maintain the structural integrity of MoS_2_ while enhancing its surface area and electrochemical performance, thereby making it a promising material for advanced electronic and optoelectronic applications ranging from energy storage to sensing devices under ambient conditions.

## 1. Introduction

Transition metal dichalcogenides (TMDs) are very promising layered materials that have been widely studied for their scalability and thickness-dependent properties [1,2,3,4,5,6,7,8,9]. TMDs are compounds with the chemical formula MX_2_, where M stands for a transition metal element (Mo, W, Nb) and X indicates a chalcogen (e.g., Se, S, Te). Bulk TMDs have a layered crystal structure assembled by stacking together two-dimensional (2D) X-M-X sheets with weak van der Waals (vdW) forces acting between adjacent layers that can be exfoliated/synthesized to convert layered crystals into 2D nanosheets by means of several techniques and methods [8,9,10,11,12,13,14,15,16,17,18,19,20,21,22,23,24,25,26]. In particular, liquid-phase exfoliation (LPE) has been proven to be a very reliable method to obtain nanosheets in liquid media from bulk via intercalation into interlayers, weakening the interlayer bonding, especially if combined with microwave radiation [16,17,27,28]. Among the TMD materials, molybdenum disulfide (MoS_2_), which is found abundantly in nature in the form of molybdenite, is one of the most studied [5,19,29,30]. Its crystal structure consists of an hexagonal plane of Mo atoms sandwiched by hexagonal planes of S atoms. These triple planes—a monolayer of MoS_2_—stack on top of each other with strong covalent bonds between the Mo and S atoms and weak van der Waals forces holding layers together, until the bulk structure is formed. Bulk MoS_2_ is reported to have an indirect bandgap of 1.2 eV— similarly to silicon—whereas 2D monolayer MoS_2_ nanosheets have a direct bandgap of 1.8 eV, possessing intriguing optical properties suitable for several applications, such as light-emitting diodes, transistors, batteries, and photovoltaics [4,10,18,22,27,28,31,32,33,34,35,36,37,38,39,40,41,42]. In fact, studies of thin and ultrathin films produced with various techniques, starting from exfoliated two-dimensional materials or bulk materials, to create devices and heterostructures that can be used in fields ranging from the medical to the energetic areas are increasingly widespread and frequent [9,12,14,22,23,43,44,45,46]. In particular, the study and the realization of third-generation photovoltaic cells that exploit the properties of 2D materials are spreading more and more. For example, 2D vdW materials have been employed to improve utilizing photoexcited hot carriers (HCs)—high-energy carriers beyond the band edges of the semiconductor material used, which are not in thermal equilibrium with the lattice—for light-harvesting applications such as hot carrier solar cells (HCSCs), enhancing the solar to electric power conversion efficiency (PCE) [47,48,49,50,51].

In this frame, a 2D MoS_2_ semiconductor material is considered very promising for its excellent optical absorption property and fast charge carrier dynamics, and, consequently, it seems natural how important it is to define solid crystal structures distinguishing between the crystalline phases of our bulk and exfoliated material.

**Figure 1:**
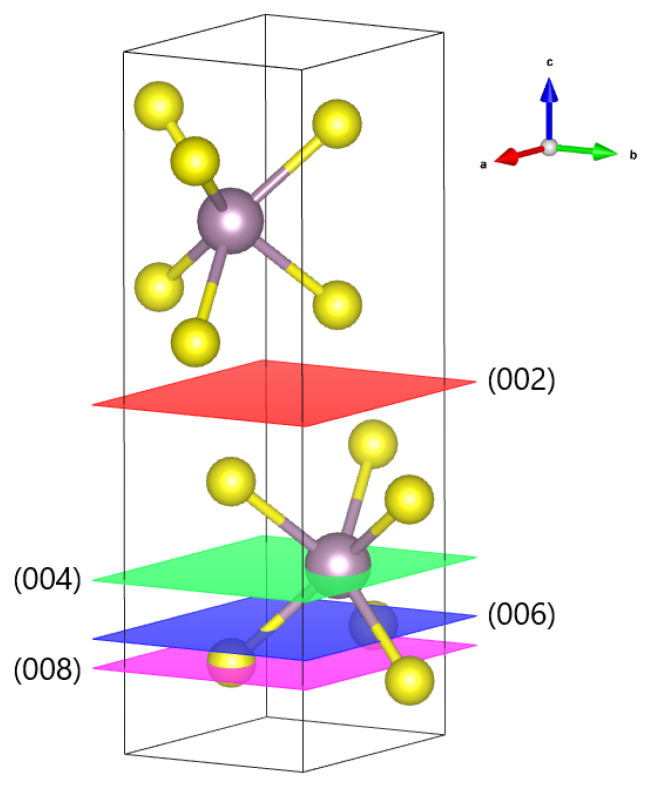
Ball-and-stick schematic model of the 2H phase unit cell for MoS_2_ reporting the parallel lattice planes and the corresponding Miller indices along the c axis. This figure is achieved by means of VESTA (Visualization for Electronic and STructural Analysis), a 3D visualization software package for structural models, volumetric data such as electron/nuclear densities, and crystal morphologies [57].

Specifically, bulk molybdenum disulfide crystals are characterized by three different polytypes, 1T, 2H, and 3R phases, where the number indicates how many monolayers are considered internally to the unit cell of the crystal lattice, and the letters T, H, and R refer to the trigonal, hexagonal, and rhombohedral structural symmetry, respectively [2,52]. The 2H phase is shown in Figure 1 as a ball-and-stick representation in which Mo and S atoms are reported in grey and yellow, respectively. Structural phases depend on the stacking arrangement of layers in MoS_2_ with respect to Mo coordination. Concerning 2D MoS_2_, five polymorphs—1H, 1T, etc.—have been suggested for the atomic structure of monolayers [2,10,52,53,54]. Then, while the 2H configuration possesses an atomic stacking sequence, a 1H arrangement denotes a single-layer structure of a 2H phase [2,9,54,55]. The structure of 2H-MoS_2_ facilitates having several exposed surfaces, one along the edges of SxMoxS layer via the c-axis named the (002) basal plane, while the latter is along the edges of SxMo and is known as the (100) plane [54]. Thus, efficiently distinguishing the presence of a monolayer or multiple monolayers and their respective crystal structure can greatly affect the creation of a device and its characteristics. The most used technique to determine the different phases of bulk and exfoliated materials is X-ray diffraction (XRD) analysis [56].

In this frame, herein we propose a study regarding the X-ray characterizations of exfoliated MoS_2_ produced by means of microwave-assisted LPE for the future realization of devices (e.g., HCSC). LPE is fairly well studied, but still there are not many papers in which this technique is assisted by microwaves, especially to obtain two-dimensional materials. Compared with what is present in the literature, in this study, we propose not only variations in the preparation of samples to be exfoliated and their subsequent filtration, but also an extensive study on exfoliated sample characterization by X-ray. In fact, by means of XRD, we obtain important information to distinguish structural differences between the bulk sample to be exfoliated and the exfoliated samples. Furthermore, we support our results obtained with XRD by accompanying them with X-ray photoelectron spectroscopy (XPS) measurements. This technique allows the identification of the elements that exist within a material (elemental composition) or are covering its surface, as well as their chemical state, and the overall electronic structure and density of the electronic states in the material. In our case, our scope is to confirm the success of the exfoliation procedure and that the exfoliated sample retains its original structure. In particular, as shown in Figure 1, we will focus our attention on the 2H phase of MoS_2_, investigating the correspondence of the family of (parallel) lattice planes along the c axis between the bulk and exfoliated samples.

## 2. Materials and Methods

In our experiments, we made use of model bulk MoS_2_ powder (Molybdenum (IV) sulfide, Alfa Aesar) and exfoliated samples obtained by means of microwave-assisted liquid-phase exfoliation (LPE) using a single-mode microwave (MW) synthesis system (Discover from CEM Corp., Matthews, NC, USA).

### 2.1. Preparation of Exfoliated MoS_2_

A microwave-heating-based exfoliation process in the presence of intercalated solvents was used for exfoliating MoS_2_. This method is based on the use of a solvent or solvents which intercalate into the interlayer spaces of the nanomaterial and reduce the attractive van der Waals interactions between the layers [15]. A microwave-assisted approach to exfoliate MoS_2_ was performed using two methods reported in the literature which involve a pre-treatment of the powder with a wet-grinding procedure using high affinity solvents to avoid powder agglomeration to achieve uniform suspensions [16,17].

#### 2.1.1. Exfoliation of MoS_2_ with NMP Solvent (MoS_2_-NMP)

The first exfoliation method used was carried out using 1-Methyl-2-pyrrolidinone (NMP), both as a pre-wetting solvent and as a solvent capable of dispersing the exfoliated MoS_2_, and then the sealed vessel containing the powder and fluid media was placed into a single-mode microwave reactor and irradiated at 140 W/60 min. After microwave irradiation, the exfoliated sample of MoS_2_-NMP was obtained by diluting the suspension with Dimethyl sulfoxide (DMSO) and filtering it with a 0.2 μm polytetrafluoroethylene (PTFE) filter membrane on a sand core filtration system. Then, the resulting residue on the filter was redispersed in ethanol, centrifuged at 5000 rpm/25 min, and the collected supernatant was evaporated under vacuum. The detailed procedure followed to produce the sample MoS_2_-NMP is reported below:

- Pre-treatment: An amount of 10.2 mg of bulk MoS_2_ powder and 0.2 mL of NMP were first sonicated for 5 min and then transferred into a 10 mL quartz vessel with another 2.8 mL of solvent.
- Microwave exfoliation: MW irradiation with the CEM Discover system and high-speed stirring to homogenize the whole reaction were used. The ‘Power Max’ function was used at 140 W for 60 min (as operation parameters) with a max temperature of 200 °C, and during microwave irradiation, the temperature was maintained at approximately 100 °C through an air-cooling system.
- Post-Treatment: After cooling, the obtained suspension was first diluted with 5 mL of DMSO, and then filtered with a 0.2 μm PTFE filter membrane on a sand core filtration system; subsequently, ethanol was used to rinse the dust deposited on the PTFE filter. The solid residue was redispersed in ethanol by 5 min of ultrasonic agitation and centrifuged with 5000 rpm/25 min to remove the thick sheets. The supernatant was then dried under vacuum at 40 °C.

#### 2.1.2. Exfoliation of MoS_2_ with ACN and NMP Solvents (MoS_2_-ACN-NMP)

The MoS_2_-ACN-NMP sample was obtained by the microwave irradiation of MoS_2_ powder pre-wetted with ACN and dispersed in NMP. Microwave irradiation was performed using an open vessel option by inserting the flask equipped with a refrigerant directly into the reaction cavity at 300 W/12 min. The green-dark suspension was centrifuged with 2000 rpm/20 min, the supernatant was filtered with a 0.2 μm PTFE filter membrane on a sand core filtration system, and an ACN/ethanol mixture was used to rinse the dust deposited on the PTFE filter. The resulting filtered solution was collected and evaporated under reduced pressure at 40 °C to obtain MoS_2_-ACN-NMP sample. In the following passages, details of the exfoliation method are given:

- Pre-treatment: An amount of 30.2 mg of bulk MoS_2_ powder and 0.5 mL of ACN were sonicated in a glass vial for about 10 min at 30 °C (until most of the solvent had evaporated).
- Microwave exfoliation: The residue pre-wetted with ACN was transferred to a 100 mL round button flask and 30 mL of MNP was added. Microwave irradiation with a single-mode microwave synthesis system and high-speed stirring was performed using an open vessel option by inserting the flask equipped with refrigerant directly into the reaction cavity of the Discover CEM microwave. The ’Power Max’ function was used at 300 W for 12 min (as operation parameters) with a max temperature of 150 °C.
- Post-Treatment: After cooling, the green-dark obtained suspension was centrifuged with 2000 rpm/20 min to remove the thick sheets. The supernatant was then filtered with a 0.2 μm PTFE filter membrane on a sand core filtration system, and an ACN/ethanol mixture was used to rinse the dust deposited on the PTFE filter. The resulting filter solution was collected and evaporated under reduced pressure at 40 °C.

### 2.2. Morphology Characterization

**Figure 2:**
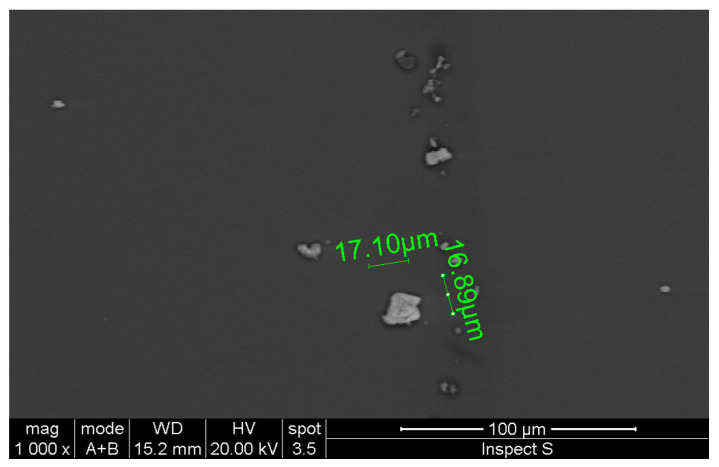
SEM acquisition of our exfoliated sample, showing lateral dimensions of almost 17 μm.

The scanning electron microscopy (SEM) image reported in Figure 2 shows an example of our exfoliated sample. The measurement was performed with a Phenom Prox (PhenomWorld)—an all-in-one desktop SEM for imaging and analysis with a fully integrated energy-dispersive spectrometer (EDS)— by using a magnification of 1000× and a working distance (WD) of 15.2 mm. As shown in the figure, we obtained lateral dimensions of almost 17 μm. Furthermore, by EDS measurements, we verified the stoichiometry of our sample, confirming the presence of sulfur and molybdenum.

### 2.3. XRD Analysis

The prepared samples were characterized by X-ray diffraction (XRD) to determine any modifications in the crystal structure of the exfoliated material, with a particular focus on 2H phase. The samples, in the final formulation, consisted of the dispersion of 1.6 mg/mL of exfoliated material in a [1:1] water/ethanol solution. After the sample was briefly vortexed, about 200 μL of such a solution was spread on a [100] Si wafer substrate by the drop-cast method, and used for the measure after complete drying.

The XRD measurements were collected on a Bruker D2 Phaser diffractometer, a benchtop instrument working in Bragg–Bentano geometry with an accuracy of about 0.02° throughout the entire measuring range. This kind of instrument is usually used for routine phase identification and quantitative phase analysis. The X-ray source is a Cu standard sealed ceramic tube with Kα at 1.5400 Å(K$\alpha_{1}$) and 1.54439 Å(K$\alpha_{2}$), filtered by a 0.5 mm Ni slab and collimated by a 0.25 mm Soller slit. The diffracted beam was recorded by a Lynxeye detector in the 1D mode, after collimation with a 0.6 mm slit and a knife-edge of 1 mm. The data were recorded from 10° to 75° with 3 s of integration time giving a total run time of about three hours. In this configuration, the Bremsstrahlung contribution is totally removed, but the K$\alpha_{2}$ is only partially filtered. As a consequence beyond an obvious intensity increment, the presence of two close wavelengths helps to identify faint signals that originated from the sample, which will always be present as doublets, considering that in this kind of application, the sample contribution is expected to be very small compared to the background generated by the sample supporting structure. Moreover, we have experienced that removing the K$\alpha_{2}$ contribution via software routines results in distorted K$\alpha_{1}$ peaks in the case of signal with low counts.

The acquired data were analyzed and visualized using Gnuplot, a open-source portable command-line driven graphing utility.

### 2.4. XPS Analysis

PHI Versa Probe II (Physical Electronics), equipped with an Al Kα (1486.6 eV) X-ray source, measured the XPS spectra. The survey spectra were recorded with an analyzer energy path of 117 eV, while the C1s, Mo3d, and S2p core levels were measured at 23.5 eV passing energy. The X-ray beam size was 100 microns at 25 W. A charge neutralization procedure was performed by simultaneous irradiation of samples using a low-energy electron beam and an ion beam before measuring the spectra. The XPS peak position was referenced to a Au metal foil (84.0 eV). XPS peaks were analyzed by using the Multipack Data Reduction Software (https://www.phi.com/surface-analysis-equipment/system-software/multipak-data-reduction-software.html, ULVAC-PHI, Inc., Chigasaki, Japan), employing a Shirley background curve. After the analysis, we made use of Gnuplot to plot the data.

## 3. Results and Discussion

### 3.1. XRD Measurements

**Figure 3:**
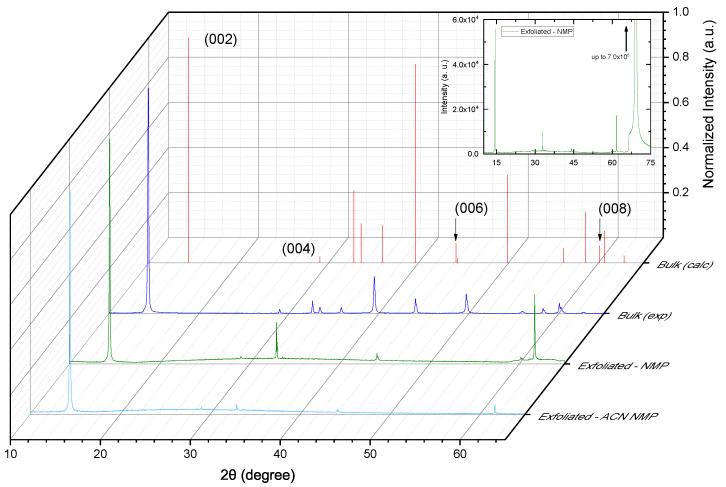
XRD spectra for calculated bulk 2H-MoS_2_ [Bulk (calc), red vertical lines], raw bulk MoS_2_ powder [Bulk (exp), blue line], MoS_2_-NMP [Exfoliated-NMP, green line] and MoS_2_-ACN-NMP [Exfoliated-ACN-NMP, light-blue line]. The inset shows the presence of an intense Si peak at about 69°, which is not reported in the 3D graph to keep the normalized peaks of the MoS_2_ samples well visible.

A panoramic view of the collected diffractograms is presented in Figure 3, in which we reported the spectra acquired for the MoS_2_ samples investigated (bulk powder and exfoliated) along with the diffraction pattern calculated for bulk 2H-MoS_2_ by means of VESTA using the corresponding CIF file. The expected Miller indices estimated in this latter case are shown in an impulse graph as red vertical lines named Bulk (calc); the blue line represents the experimental XRD data for our raw bulk MoS_2_ powder, named Bulk (exp); and the green and light-blue lines refer to the XRD spectra of exfoliated MoS_2_-NMP and MoS_2_-ACN-NMP samples, named Exfoliated-NMP and Exfoliated-ACN-NMP, respectively.

As can be observed from the inset of the same figure, the diffractograms are dominated by the [100] Si contribution. That consists of the expected [400] strong peak at 69.1298, generated by K$\alpha_{1}$ and K$\alpha_{2}$, extending up to several orders of magnitude. Note that the asymmetry of this peak is due to the sharp rising edge of the absorption of the Ni filter [58]. Close to this peak, there is also the presence of a [400] contamination from the Kβ (1.39220 Å) peak at 2θ = 61.68966°. Furthermore there is also the occurrence of the basis-forbidden Si [200] reflection, which can be observed in the range between about 31° and 35°, due to multiple diffraction (Umweganregung) in the classic ω−2θ scan [59].

As for the measured samples, a first glance at Figure 3 confirms that the raw material used is consistent with the expected MoS_2_ powder diffraction pattern; moreover, in the exfoliated samples, only the peaks originated by planes parallel to *c* axes are observed, and this suggests that the exfoliation was successful and that the obtained flakes are correctly deposited on the Si surface. For a more detailed analysis of the peak position (i.e., plane spacing) in the exfoliated samples, we relayed on the [400] Si peak position, which always presents as a substrate. This peak has been fitted with the sum of two lorentzians generated by the two Kα line reflections by the [400] plane (d = 1.35765Å) at a 2θ of 69.123 and 69.321, respectively. This was performed in order to determine both the instrumental angular shift and the intensity ratio among Kα lines. Those values were then used to determine the position of the remaining peaks by means of a fitting procedure that includes both CuKα lines. The experimental 2θ shift was found to be of the order of (7–8$\cdot 10^{-3})$°, which is below instrumental resolution, while for the Kα intensity ratio, we obtained 0.46–0.50.

The obtained results are listed in Table 1, in which we report the Miller indexes and the corresponding interplane distances obtained from the literature (CIF data) along with the experimentally found values for the bulk powder sample and the two types of exfoliated samples.

**Figure 4:**
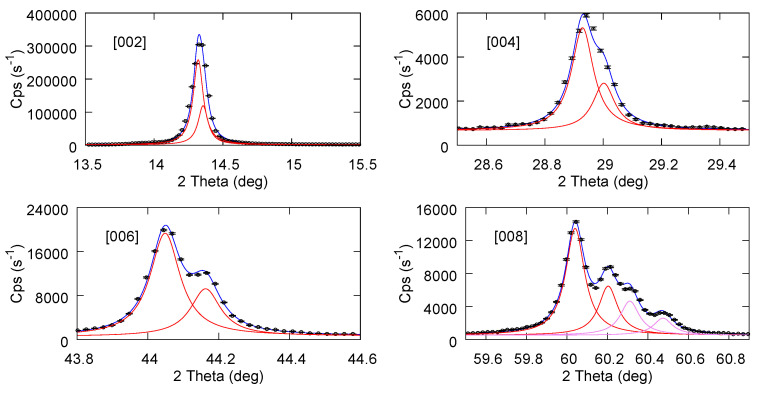
Experimental data and fitted lorentzian doublet for MoS_2_ powder.

**Figure 5:**
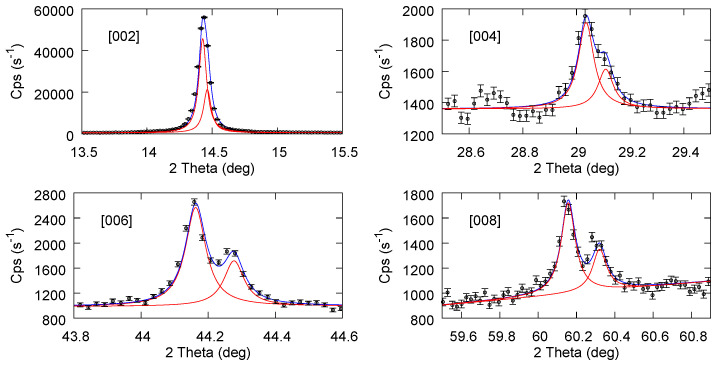
Experimental data and curve fitting of K$\alpha_{1}$ and K$\alpha_{2}$ for MoS_2_ exfoliated using NMP solvent.

**Figure 6:**
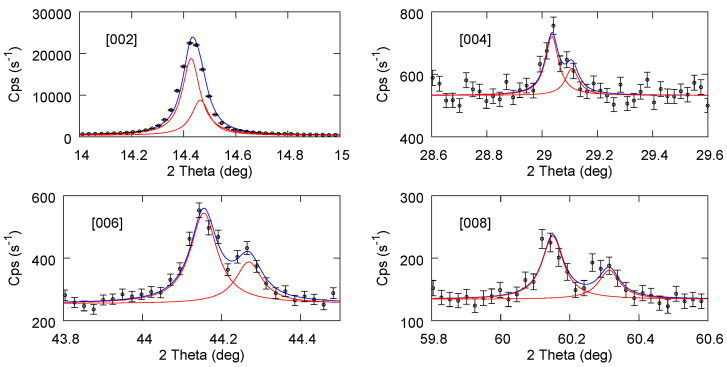
Experimental data and curve fitting of K$\alpha_{1}$ and K$\alpha_{2}$ for MoS_2_ exfoliated using ACN-NMP solvent.

Figure 4 reports the fit results for the starting bulk powder sample partly mixed with Si powder (in order to use the [400] Si peak as a reference for accurate calibration). Only the [00n] planes from MoS_2_ are considered, since only these are observed after the exfoliation processes. In particular, for the last sub figure, a second component has been added to also include the [112] plane of MoS_2_, which falls close to [008]. In Figure 5 and Figure 6, we report the fit results for exfoliated samples using NMP and ACN-NMP as the solvent in the production process. The fitting procedure and the parameters obtained are given in the Supplementary Information. Although the statistical error is generally bigger than in the bulk powder case, the quality of the fits nevertheless allows a reliable assignment of the peak positions and the consequent inter plane distances.

### 3.2. XPS Measurements

**Figure 7:**
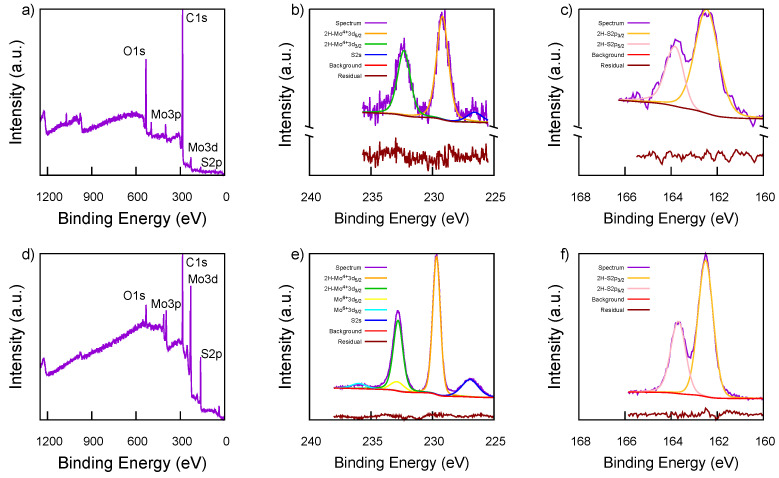
Deconvoluted XPS for exfoliated MoS_2_ (**a**) survey, (**b**) Mo3d, (**c**) S2p and bulk MoS_2_, (**d**) survey, (**e**) Mo3d, and (**f**) S2p.

The surface characteristics of all the samples were investigated using X-ray photoelectron spectroscopy (XPS). The survey spectra of the bulk MoS_2_ (Figure 7d) indicate that the samples primarily contain C, Mo, S, and O elements.

The surface composition in weight percentage together with the atomic ratio S/Mo are reported in Table 2 for both samples investigated. The nearly doubled percentage of carbon and the traces of Na (about 1.80 wt %) observed in the survey of the exfoliated MoS_2_-ACN-NMP sample (Figure 7a) are likely related to the solvent used.

The high-resolution Mo3d spectrum of the bulk MoS_2_ shown in Figure 7e reveals two main doublet peaks corresponding to the Mo3$d_{5/2}$ and Mo3$d_{3/2}$ states, located at binding energies around 229.67 eV and 232.80 eV, which are consistent with M$o^{4+}$ species. Additionally, small doublet peaks at 232.85 eV and 235.98 eV indicate the presence of an oxidized M$o^{6+}$ species. The Mo3$d_{5/2}$-Mo3$d_{3/2}$ splitting energy is 3.13 eV. The narrow full-width-at-half-maximum (FWHM) of 0.73 suggests the presence of the 2H phase (hexagonal crystal structure) according to the literature [60]. The spectrum also displays a peak for S2s at 226.95 eV, which was included in the fitting with the aim to have a good match between fitted curves and raw data, as evidenced by the fitting residual. Similarly, the S2p spectrum in Figure 7f exhibits only the 2H phase with a strong doublet peak at binding energies of 162.50 eV for S2$p_{3/2}$ and 163.69 eV for S2$p_{1/2}$, with a splitting energy of 1.18 eV, typical for metal sulfides [61,62]. The Mo3d spectra of exfoliated MoS_2_ (Figure 7b), is slight shifted towards lower binding energies. The deconvoluted spectra reveal the peaks at 229.19 eV for M$o^{4+}$ 3$d_{5/2}$ and 232.32 eV for M$o^{4+}$ 3$d_{3/2}$ [63], confirming the presence of the 2H phase. The modest shift observed in the case of Mo3d can be attributed to the reduction in the oxidation state of Mo, supported by the disappearance of the ${\text{Mo}}^{6+}$ component after the exfoliation treatment [64]. The S2p spectra (Figure 7c) for exfoliated MoS_2_ closely resemble those of the bulk sample, displaying a doublet peak at 163.35 eV and 162.15 eV, which correspond to the 2H-S2$p_{1/2}$ and 2H-S2$p_{3/2}$ states, respectively [63]. The Mo/S stoichiometry was also analyzed using XPS data, revealing a sulfur-to-molybdenum ratio of about 2.04 based on the atomic concentration obtained by correlating the respective areas (see Table 2). This stability confirms that the exfoliated sample maintains its original structure, as also reported by other authors [17].

## 4. Conclusions

The aim of this study was to characterize exfoliated MoS_2_ samples, produced by means of microwave-assisted liquid-phase exfoliation (LPE) from bulk powder in 1-methyl-2-pyrrolidone (NMP) or ACetoNitrile (ACN) + 1-methyl-2-pyrrolidone (NMP) solvents, to reveal the correct exfoliation of the sample and demonstrating, in addition, the preservation of the 2H phase after exfoliation with only the retention of the [00n] planes. In fact, as shown in Figure 3, the XRD peaks of our exfoliated samples—compared with the bulk ones—showed a 2H phase and the loss of lattice plans outside the [00n] ones, causing us to believe that only very few layers overlap and the samples are well flattened along the z-axis. Furthermore, as shown in Table 1, which reports the values of the lattice distances obtained by our data analysis with a precise curve fitting procedure, there are only differences in thousandths of Angstrom between all the investigated samples. Furthermore, the XPS analyses on bulk and exfoliated samples support our XRD results, showing the success of the exfoliation procedure and that the exfoliated sample retains its original structure. In this frame, the low-dimensionality and the crystal phase of our exfoliated samples allows us to state that they can be exploited to produce coatings for their use in future energy applications, such as HCSCs.

## Figures and Tables

**Table 1:** Lattice distances in Angstrom.

Miller Index ^1^	CIF Data	Powder	NMP	ACN-NMP
[002]	6.1500	6.14348	6.16374	6.15999
[004]	3.0750	3.07439	3.07866	3.07807
[006]	2.0500	2.04923	2.05103	2.05105
[008]	1.5375	1.53672	1.53758	1.53769

^1^ Crystal planes refer to the 2H phase typical of the bulk.

**Table 2:** Surface composition in percentage by weight and atomic ratio S/Mo for powder and exfoliated MoS_2_.

Sample	O1s (wt %)	C1s (wt %)	Mo3d (wt %)	S2p (wt %)	Atomic Ratio S/Mo
powder	6.67 ± 0.21	40.02 ± 0.13	31.42 ± 0.18	21.90 ± 0.15	2.04 ± 0.22
exfoliated	16.00 ± 0.22	78.62 ± 0.11	3.18 ± 0.26	2.20 ± 0.27	2.00 ± 0.24

## Data Availability

The original contributions presented in the study are included in the article, further inquiries can be directed to the corresponding author.

## Supplementary Materials

The following supporting information can be downloaded at: https://www.mdpi.com/article/10.3390/ma17163887/s1.

## Abbreviations

The following abbreviations are used in this manuscript:

TMD	transition metal dichalcogenides
2D	two-dimensional
vdW	van der Waals
LPE	liquid-phase exfoliation
MoS_2_	molybdenum disulfide
HC	hot carrier
HCSC	hot carrier solar cell
XRD	X-ray diffraction
XPS	X-ray photoelectron spectroscopy
MW	microwave
NMP	1-methyl-2-pyrrolidone
DMSO	Dimethyl sulfoxide
PTFE	Polytetrafluoroethylene
ACN	Acetonitrile
SEM	scanning electron microscopy
EDS	energy-dispersive spectrometer
VESTA	Visualization for Electronic and STructural Analysis
PTFE	Polytetrafluoroethylene
