# Supplementary material for: X-ray Characterizations of Exfoliated MoS2 Produced by Microwave-Assisted Liquid-Phase Exfoliation
Source: Materials (Basel). 2024 Aug 6;17(16):3887. doi: 10.3390/ma17163887 (PMC11355266; doi:10.3390/ma17163887)
Supplement: Supplementary file 1 [file materials-17-03887-s001.zip › materials-3128950-supplementary.pdf]

X-ray characterizations of exfoliated MoS_2 produced by microwave-assisted liquid phase exfoliation - Supplementary Information -

2024–08–01

Fitting strategy

In XRD diffractograms the contribution of the exfoliated sample is very low compared to Si support and the determination of MoS_2 peaks position can be challenging. Moreover it is important to ascertain the presence of likely angular instrumental shift as function of reflection angle (2θ). To this aims we have used Si as internal standard in our measurements on MoS_2 and we have fitted the experimental data including both Kα_1 and $K\alpha_2$ since the a priori correction routines have proved to be unsatisfactory.

For a detailed analysis of XRD peaks position in the exfoliated samples we relayed on the [400] Si reflection with d=1.357735 and 2θ = 69.32109, that is presents with high intensity in (100)Si wafer that we have used as support for drop-casting of exfoliated MoS_2 in water-ethanol solution.

The analysis of XRD diffractograms of Bulk MoS_2 powder was instead performed by mixing it with Si powder in equal quantities. In this case we relayed on the [111] , [220] and [311] Si reflection with d=3.135555, 1.920128, 1.637490 and 2θ = 28.44228, 47.30261, 56.12219 respectively. In this cases the well known peaks position of Si reference allowed for an accurate determination of a 2θ dependent instrumental shift and a precise evaluation of K$\alpha_2/K\alpha_1$ intensity ratio.

In all the considered reflections doublet the fitting function vs. 2θ has the general form of two Lorentzian plus a linear background:

$$\begin{aligned} Fit(x) =& A\big(L1(x) + K_{ratio}L2(x)\big) + bkg(x) \qquad \text{(S1)} \\ L1(x) =& \frac{\Gamma}{\pi(x - 2\theta'_{hkl})^2 + \Gamma^2)} \\ L2(x) =& \frac{\Gamma}{\pi(x - 2\theta''_{hkl})^2 + \Gamma^2)} \\ bkg(x) =& mx + c \end{aligned}$$

with

$$x = 2\theta$$

$$2\theta_{hkl} = \arcsin(\lambda/2d_{hkl}) \quad \text{from the Bragg law.}$$

In equations 1: A is the total intensity and K_{ratio} is $K\alpha_2$, $K\alpha_1$ ratio, $2\theta'_{hkl}$ and $2\theta''_{hkl}$ are the reflections of [hkl] planes produced by the two $K\alpha$ characteristic line of Cu anode (λ_1=1.540560, λ_2=1.5444256) and Γ is the shared lorentian half width at half maximum (hwhm).

In the actual fitting the free parameters were the total intensity A, the [hkl] planes spacing d and the hwhm Γ besides the slope m and intercept c of the linear background. The K_{ratio} being previously determined by fitting reference peaks in Si where the [hkl] planes spacing d is well known. K_{ratio} is found to be 0.4599, which is slightly lower then the canonical values of 0.50, due to the use of Ni filter.

The parameters of the fits performed are given below by referring to the figures in the main text.

Bulk MoS_2

[hkl]	A	d	hwhn	shift
002	16087 (2.77%)	6.1435 (0.0101%)	0.038 (4.51%)	-0.026
004	309.77 (10.24%)	3.07439 (0.0175%)	0.046 (17.60%)	-0.0374
006	940.71 (1.13%)	2.04923 (0.0017%)	0.039 (4.51%)	-0.0496
008	2159.41 (2.17%)	1.53672 (0.0050%)	0.045 (3.02 %)	-0.0888
112	697.39 (4.67%)	1.53127 (0.0071%)	0.044 (3.26%)	-0.0887

MoS_2-NMP

[hkl]	A	d	hwhn	shift
002	5166.7 (1.72%)	6.16374 (0.0056%)	0.036 (2.90%)	0.0698
004	59.29 (9.73%)	3.07866 (0.0162%)	0.034 (17.56%)	0.0580
006	190.89 (2.70%)	2.05103 0.0035%)	0.038 (5.042%)	0.0459
008	98.16 (14.97%)	1.53758 (0.0121%)	0.044 (15.89%)	0.0330

MoS_2-ACN-NMP

[hkl]	A	d	hwhn	shift
002	2392.99 (1.48%)	6.15999 (0.0052%)	0.0413 (2.42%)	0.0533
004	16.6636 (14.15%)	3.07807 (0.0208%)	0.027 (29.89%)	0.0416
006	37.6194 (5.33%)	2.05105 0.0070%)	0.0412 (9.73%)	0.0294
008	13.9401 (23.74%)	1.53769 (0.0167%)	0.040 (19.79%)	0.0165
